# Basilar Artery Tortuosity Is Associated With White Matter Hyperintensities by TIMP-1

**DOI:** 10.3389/fnins.2019.00836

**Published:** 2019-08-14

**Authors:** Dao Pei Zhang, Yan Fang Peng, Huai Liang Zhang, Jian Gong Ma, Min Zhao, Suo Yin, Tian Tian Wei

**Affiliations:** ^1^Department of Neurology, The First Affiliated Hospital of Henan University of Chinese Medicine, Zhengzhou, China; ^2^Department of Neurology, The Fifth Affiliated Hospital of Sun Yat-sen University, Zhuhai, China; ^3^Department of Image, People’s Hospital of Zhengzhou Affiliated to Southern Medical University, Zhengzhou, China; ^4^Clinical Medical Testing Center, People’s Hospital of Zhengzhou Affiliated to Southern Medical University, Zhengzhou, China

**Keywords:** matrix metalloproteinases, tissue inhibitor of metalloproteinase-1, vertebrobasilar dolichoectasia, cerebral small vessel disease, white matter hyperintensities

## Abstract

**Background and Purpose:**

To test the hypothesis that the imbalance between matrix metalloproteinases (MMPs) and tissue inhibitor of metalloproteinases (TIMPs) may play a potential role in bridging vertebrobasilar dolichoectasia (VBD) with lacunar infarction (LI) and white matter hyperintensities (WMH).

**Methods:**

We studied 212 patients with vertigo who underwent multimodal magnetic resonance imaging (MRI) tests for VBD, LI, and WMH identification. We investigated biomarkers of VBD with magnetic resonance angiography (MRA) via various physical characteristics of the vertebrobasilar arteries (VBAs). Similarly, LI and WMH biomarkers were extracted using T2-weighted and fluid attenuated inversion recovery (FLAIR) images. We first determined which of these neuroimaging markers were significant identifiers of VBD, LI and the different grades of WMH. We then sought to draw potential mechanistic conclusions from these MRI-derived parameters, by associating the aforementioned biomarkers with MMP and TIMP serum levels in patient blood samples using non-parametric statistical tests.

**Results:**

MMP-9 serum level was significantly higher in vertigo patients with VBAs dilation and basilar artery (BA) elongation compared to those with healthy arterial size, and the ratio of MMP-9/TIMP-1 level were higher in those patients. TIMP-1 level was also markedly higher in vertigo patients with BA tortuosity than those without BA tortuosity. The bending length (BL) of the BA was positively correlated with TIMP-1. The length, BL, and tortuosity index of the BA, as well as serum levels of TIMP-1 were greater in patients with higher WMH grades compared to those with low WMH grades. The vertebral artery and BA diameters, and the levels of MMP-2, -3, -9, TIMP-2 and cathepsin L were similar in patients with different WMH grades.

**Conclusion:**

In vertigo patients, we found various probably associations between MMP-9 and TIMP-1 with arterial alterations linked to both VBD and WMH that may help with the diagnosis and treatment of such diseases in the future.

## Introduction

Vertebrobasilar dolichoectasia (VBD) is a clinical dysfunction characterized by an elongated, dilated and/or tortuous vertebral artery (VA) and/or basilar artery (BA) (Gutierrez et al., 2011; Yuan et al., 2014; Zhai et al., 2018). The prevalence of VBD is approximately 0.08–6.5% in the general population, while in patients with stroke, the prevalence ranges from 3 to 17% (Pico et al., 2015; Samim et al., 2016; Del Brutto et al., 2017). Similarly, cerebral small vessel disease (CSVD) is a heterogeneous dysfunction affecting the perforating cerebral arterioles, capillaries or venules. CSVD may manifest as acute focal neurological symptoms, dementia, gait disturbance and progressive cognitive impairment, however, the majority of patients are asymptomatic for large periods of time (Wardlaw et al., 2019). Nevertheless, while behavioral indicators may not be a reliable source of detecting VBD or CSVD, recent advances in magnetic resonance imaging (MRI) technology has led to the identification of various neuroimaging markers of both disease states (Pico et al., 2015; Del Brutto et al., 2017). The robustness and widespread accessibility of acquiring magnetic resonance angiography (MRA) information allows for simple quantification of vascular descriptors, such as the length and diameter of arteries linked to VBD. Alternatively, anatomy-based MRI scans can identify lacunar infarcts (LI), white matter hyperintensities (WMH), dilated perivascular spaces, cerebral microbleeds and brain atrophy that are all regarded as indicators of CSVD (Norrving, 2015). Thus, in the current study, we employed various cutting-edge MRI techniques to identify and classify VBD and CSVD disease states in a large patient population.

It has been hypothesized that the involvement of matrix metalloproteinases (MMPs) and tissue inhibitor of metalloproteinases (TIMPs) play a meaningful role in both VBD and CSVD since both dysfunctions largely affect arterial media (Gutierrez et al., 2011; Pico et al., 2015; Del Brutto et al., 2017). In particular, VBD has been reported to be associated with elevated levels of MMP-9 expression alone or combined with low levels of TIMP-2 expression among individuals with HIV infection (Gutierrez et al., 2016). In a study using a mouse model focusing on differential inter-strain susceptibility to VBD, dolichoectasia was also reported to be associated with high MMP-12 and MMP-9 (Zhu et al., 2017). On the contrary, in a prospective cohort study of stroke patients, dolichoectasia was reported to be connected to a lower plasma level of MMP-3 (Pico et al., 2010). As for CSVD, a brain-autopsy study revealed that microglia/macrophage cells containing MMP-3 were more often present around small perforating arteries and areas of severe white matter damage corresponding to leukoaraiosis (Rosenberg et al., 2001). A recent study that genotyped MMP-2-1306 T/C and MMP-9-1562 C/T in a relatively large cohort of CSVD patients and controls came to a similar conclusion that MMP-2-1306 T/C polymorphism was associated with moderate or severe leukoaraiosis (Zhang et al., 2015). However, the mechanism regarding how the regulation of MMPs and TIMPs plays a role in the development and progression of VBD or CSVD remains poorly understood.

To investigate the role of MMPs and TIMPs in the pathogenesis of VBD and CSVD, we first used various MRI techniques to quantitatively identify significant neuroimaging biomarkers of these two neurological dysfunctions. We then extracted MMP and TIMP serum levels from blood samples to associate disease phenotypes found in the brain with the metabolic processes of the body. In this sense, we sought to draw mechanistic conclusions behind the dysfunctions of interest by associating MMP and TIMP serum levels with vertigo patients with and without VBD or CSVD.

## Materials and Methods

### Population

The study was approved by the Ethics Committee of Zhengzhou People’s Hospital and written informed consent was obtained from all participants. Data from 524 patients with vertigo, hospitalized at the Department of Neurology of Zhengzhou People’s Hospital (China) from December 2014 to May 2017, were considered for participation.

**FIGURE 1:**
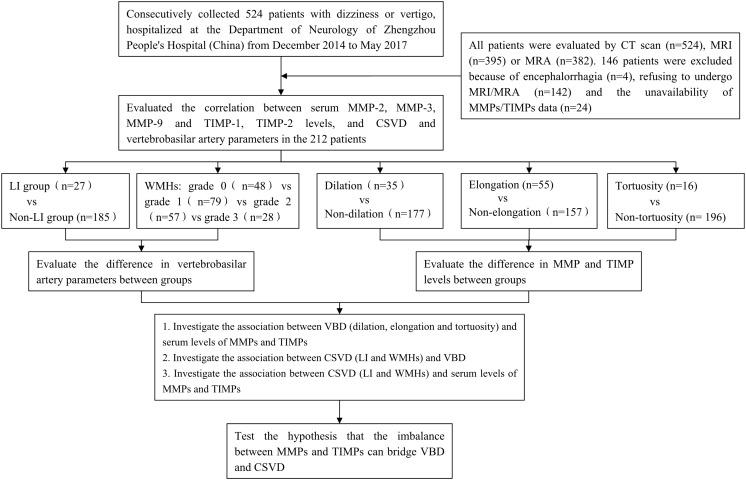
Overall study roadmap. Flow chart detailing patient recruitment, parameter extraction, and analytical methods.

According to a standard protocol, a trained neurologist (DZ) performed a neurological and vestibular examination on each patient. Most patients also underwent confirmatory caloric testing of vestibular function. Patients were included if they fulfilled the following criteria: (1) complaint of vertigo (main symptoms including spinning, swaying, nausea, vomiting and unsteady gait); (2) >18 years old; (3) exhibit at least one vascular risk factor (see detailed description below); (4) had negative results in the *Dix-Hallpike* test and the *Roll* test. Patients were excluded if they reported/exhibited one of the following: benign paroxysmal positional vertigo (BPPV), Meniere’s Disease, aural vertigo, medication/drug intoxication, recurrent ischemic strokes, atrial fibrillation or history of congenital heart disease. All patients were evaluated by CT scan (*n* = 524), MRI (*n* = 395) and/or MRA (*n* = 382). 146 patients were excluded due to encephalorrhagia (*n* = 4), the refusal to undergo MRI/MRA (*n* = 142), or the unavailability of MMP and TIMP data (*n* = 24). The 212 remaining patients with available MRI, MRA, MMPs and TIMPs participated in this study. A detailed roadmap for this recruiting process, as well as the analytical processes to compare the participating patients, is provided in Figure 1.

Various vascular risk factors were also examined and are as follows: history of arterial hypertension (previous diagnosis of arterial hypertension: systolic blood pressure >160 mmHg, diastolic >90 mm Hg, or past or present use of antihypertensive agents), diabetes mellitus (previous diagnosis of diabetes, or past or present use of antidiabetic agents), hyperlipidemia (cholesterol >5.17 mmol/L, triglycerides >1.71 mmol/L, or both), hyperhomocysteinemia (>15.0 μmol/L), drinking at least once a week (one standard alcoholic beverage is equivalent to 120 mL of wine, 360 mL of beer, or 45 mL of distilled spirits), smoking (having continuously or cumulatively smoked for more than 6 months and at least one cigarette per day), moderate or severe intracranial/extracranial arterial stenosis (the degree of artery stenosis >50%) and history of coronary artery disease (CAD). All patients with vascular risk factors had been previously diagnosed as such and/or were already taking medications for their conditions.

### Assessment of Neuroimaging Markers of Vertebrobasilar Dolichoectasia

Neuroimaging markers of VBD were evaluated using MRA images collected with a 3.0-T scanner (GE Medical, Piscataway, NJ, United States) within the first-week hospitalization. Three-dimensional time-of-flight (TOF) MRA was performed (repetition time = 24 ms, echo time = 6 ms, FOV = 24 × 24 cm and section thickness = 0.8–1.6 mm) with image reconstruction using the maximum intensity projection.

**FIGURE 2:**
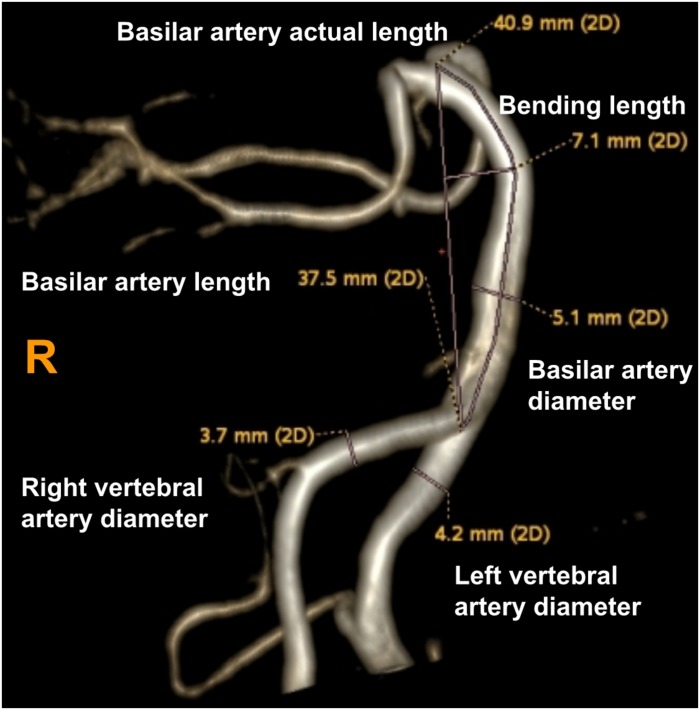
Using a volume-rendered angiogram, the basilar artery tortuosity was measured in a vertebrobasilar dolichoectasia (VBD) patient. The picture shows that the right vertebral artery diameter is 3.7 mm, left vertebral artery diameter is 4.2 mm, basilar diameter is 5.1 mm, basilar artery length (BAL) is 37.5 mm, bending length (BL) is 7.1 mm and actual length of basilar artery is 40.9 mm. Tortuosity index (TI) of the BA = (40.9/37.5 – 1) × 100 = 9.0.

**FIGURE 3:**
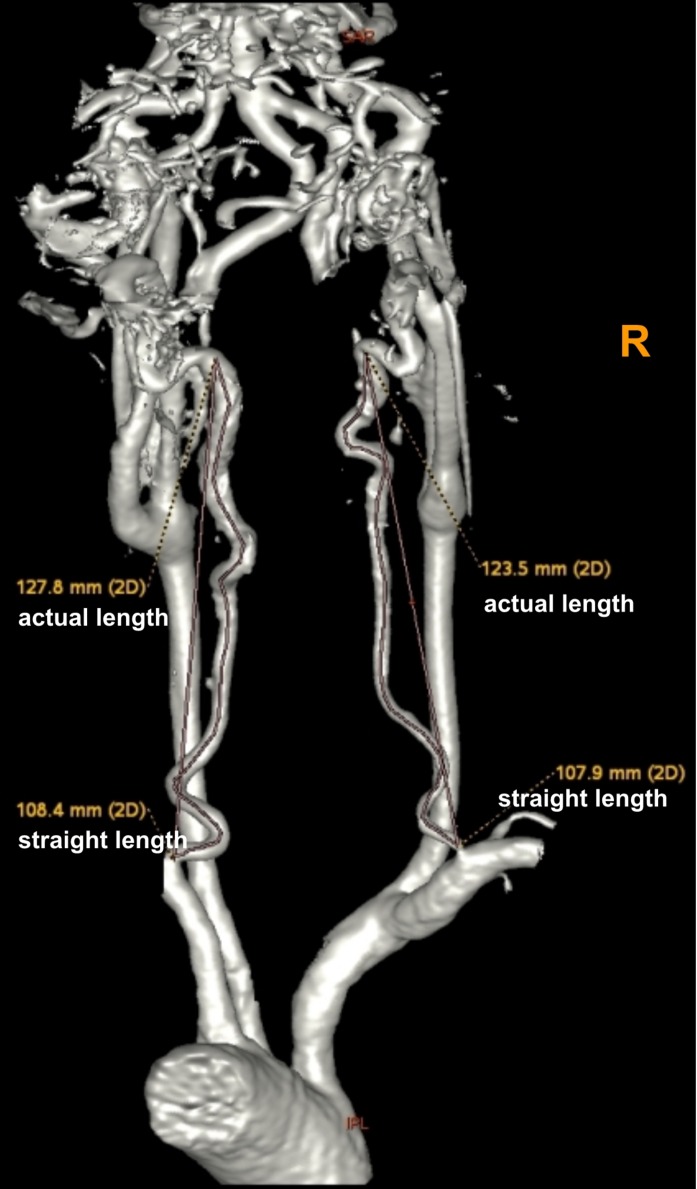
Using a volume-rendered angiogram, the vertebral artery tortuosity was measured in a vertebrobasilar dolichoectasia (VBD) patient. The picture shows that the right vertebral artery actual length is 123.5 mm, straightened length is 107.9 mm, tortuosity index (TI) of the right VA = (123.5/107.9 – 1) × 100 = 14.5 and left vertebral artery actual length is 127.8 mm, straightened length is 108.4 mm, tortuosity index (TI) of the left VA = (127.8/108.4 – 1) × 100 = 17.9.

Using these MRA images, we extracted the maximum diameter and tortuosity index (TI) of the BA and VAs, the basilar artery length (BAL) and the bending length (BL) of the BA. MRI analysis was performed by two experienced radiologists (SY and MY) blinded to clinical and demographic data. VA diameters were measured at three consecutive points, 3 mm apart, starting from the bilateral VA junction (only the maximum value was recorded for further analysis). The BA diameter was measured at the mid-pons level on TOF source images. BAL refers to a standard line length drawn from the top of the BA to the junction of both VAs (namely, the straightened length of BA), the actual length of BA was measured by tracing the course of the vessel from the top of the BA to the junction of both VAs. The BL refers to the vertical length between the midpoint of the width of the BA at the point of greatest bending and the standard line (Nishikata et al., 2004; Figure 2). For both VAs, the actual length was measured by tracing the course of the vessel from its origin to the vertebral level of C2, and the straightened length was calculated by measuring the linear distance from the origin to the end of the vessel (Morris et al., 2011; Figure 3). The TI of the BA and VAs was defined according to Eq. 1.

(1) $$\left(\frac{a\,c\,t\,u\,a\,l\;l\,e\,n\,g\,t\,h}{S\,t\,r\,a\,i\,g\,h\,t\,e\,n\,e\,d\;\,l\,e\,n\,g\,t\,h}-1\right)\times 100$$

Patients were classified as VBD if they met one or more of the following criteria:

(1) diameter of the BA ≥ 4.5 mm or diameter of the VAs ≥ 4.0 mm (dilation) (Smoker et al., 1986; Zhai et al., 2018); (2) BAL ≥ 29.5 mm (elongation) (Ubogu and Zaidat, 2004); (3) the BL ≥ 8.0 mm (Thijs et al., 2017) (we also consider this condition as tortuous).

### Assessment of Neuroimaging Markers of Cerebral Small Vessel Disease

**FIGURE 4:**
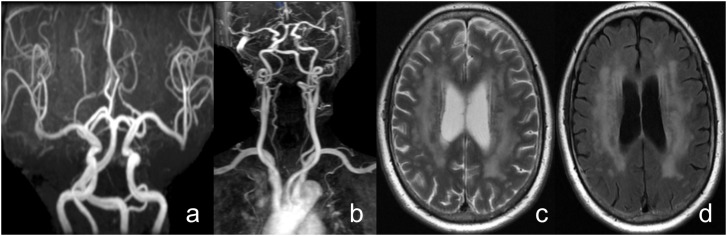
A female patient, 71 years old, with complaints of “vertigo, nausea” and a history of hypertension. **(a)** Head MRA shows vertebrobasilar artery dolichoectasia, **(b)** Neck contrast enhanced MRA shows right vertebral artery dysplasia and bilateral vertebral artery tortuosity, **(c,d)** T2WI and FLAIR shows the side body of the lateral ventricle white matter high signal, Fazekas grade 3.

Neuroimaging markers of CSVD were evaluated using conventional T2-weighted and fluid attenuated inversion recovery (FLAIR) anatomical images collected with a 3.0-T scanner (GE Medical, Piscataway, NJ, United States) within the first-week hospitalization. All images were obtained in the axial plane with voxels of size 1 mm wide × 1 mm high, × 5 mm thick (TR = 4.8 s, TE = 6 ms, field of view 320 × 320 mm). CSVD was evaluated by two experienced radiologists blinded to clinical and demographic data, following the standards for reporting MRI-based vascular changes (Wardlaw et al., 2013). LI were defined as fluid-filled cavities in the basal ganglia, brain stem, or subcortical white matter with a diameter of 3 to 15 mm. WMHs were defined as periventricular lesions appearing hyperintense on T2-weighted and FLAIR images without complete tissue destruction. WMHs were grouped into four grades according to the modified Fazekas scale (0 = absent; 1 = pencil-thin lining; 2 = halo of ≥5 mm thickness; 3 = irregular white matter hyperintensities extending into deep white matter) (Fazekas et al., 1987; Figure 4).

### Serological Test

Venous blood samples were drawn within 1 week of hospital admission. Samples were allowed to clot for 30 min at room temperature in serum separator tubes (SST) before centrifugation for 15 min at 1000 *g*. The plasma samples were frozen and stored at ≤−80°C until further use.

Matrix metalloproteinases and TIMPs concentrations were tested using commercially available quantikine enzyme-linked immunosorbent assay (ELISA) kits (SMP300/SMMP200/SMP900/STM100/DTM200, R&D Systems, Minneapolis, MN, United States). All samples were brought to room temperature before use and were analyzed in duplicate. The samples were diluted in the appropriate fold in assay dilution. A sample or standard was then added and incubated for 2 h at room temperature on a horizontal orbital microplate shaker (0.12^″^ orbit) set at 500 ± 50 rpm. This was followed by incubation for 2 h with the following antibodies conjugates: total MMP-2, human MMP-3, human MMP-9, human TIMP-1 or human TIMP-2. Substrate solution was added to the samples and was incubated for 30 min, after which a stop solution was added. The absorbance of color at 450 nm was measured using a microplate reader (Multiskan FC, Thermo) within 30 min. All steps were performed at room temperature (20–25°C). All absorbance results are expressed as nanogram per milliliter (*ng*/ml).

Cathepsin L concentration was detected with a DuoSet ELISA kit (DY008, R&D Systems, Minneapolis, MN, United States). We sought to preprocess the microplates before running the assay procedure. Diluted capture antibody was coated to the microplate and incubated overnight. The plate was later blocked by incubating it with reagent diluent for a minimum of 1 h. Since unblocked antibody was washed away, the plates were ready for sample addition. Samples or standards were added and incubated for 2 h. The detection antibody and streptavidin-horseradish peroxidase were added in sequence and incubated for 20 min. After adding substrate solution and allowing for 20 min of incubation, tetramethyl benzidine color liquid was added and at this point, a blue color developed in the microplate that was proportional to the amount of analyte present in the sample. Then, a stop solution was added, followed by gentle tapping to ensure thorough mixing, and the blue color turned to yellow. We determined the absorbance of the color immediately, using a microplate reader (Multiskan FC, Thermo) set to 450 nm. All steps were performed at room temperature (20–25°C) and results are expressed as picogram per milliliter (*pg*/ml).

### Statistical Analysis

Data were analyzed with IBM SPSS version 20.0 and *P* < 0.05 was considered significant. Demographic and clinical characteristics between VBD patients and non-VBD patients, and between CSVD patients and non-CSVD patients were respectively, evaluated by univariate analysis with a Student’s *t* test for continuous factors and a χ2 test for dichotomous factors (Fisher’s exact test was used when the expected cell frequency was <5). Significant risk factors were accounted for in the multivariate analysis with adjustment for age and gender. MMPs and TIMPs serum levels between different VBD subclassifications (dilation vs. non-dilation group, tortuosity vs. non-tortuosity group, elongation vs. non-elongation group) were respectively, evaluated with a Mann-Whitney *U* test. The correlation of serum MMPs and TIMPs levels and VBA parameters were calculated as the *Pearson* correlation coefficient. Testing of VBA parameters and serum MMPs and TIMPs levels between LI individuals and non-LI individuals was performed with a Mann-Whitney *U* test, and between patients with different WMH grades with a Kruskal-Wallis test.

## Results

### Demographic and Clinical Characteristics

Of all 212 patients with available MRA and MMP data, the mean age was 60.2 ± 12.5 and 54% were male. In terms of the VBD imaging markers, 21 exhibited dilation, 32 elongation, 1 tortuosity, 8 dilation and elongation, 9 elongation and tortuosity, and 6 all of these. As for CSVD, we identified 27 patients with LI (18 lacunes and 9 multilacunar state) and 164 patients with WMH. The classification of the periventricular lesions for WMH patients were as follows: 79 pencil-thin lining, 57 halo of ≥5 mm thickness, 28 irregular white matter hyperintensities extending into deep white matter. In vertigo patients with VBD, 43 patients were also diagnosed with CSVD, which was significantly different from those without VBD (χ^2^ = 8.033, *P* = 0.018).

In the current study, age was found to be an independent risk factor of VBD (*P* = 0.011), while gender, arterial hypertension, diabetes, dyslipidemia, hyperhomocysteinemia, smoking and alcoholism were not considered risks factors (all *P* > 0.0.5). Age, carotid arteries plaque and arterial hypertension were associated with CSVD whereas gender, diabetes, dyslipidemia, hyperhomocysteinemia, smoking and alcoholism were not associated with CSVD (Table 1).

**TABLE 1:** Demographic and clinical characteristics between VBD and non-VBD patients and between CSVD and non-CSVD individuals.

	**VBD (*n* = 77)**	**Non-VBD (*n* = 135)**	**OR (95% CI)**	**CSVD (*n* = 170)**	**Non-CSVD (*n* = 42)**	**Unadjusted, OR (95% CI)**	**Adjusted age and gender, OR (95% CI)**
Age (y), mean ± SD	64.12 ± 12.014	59.56 ± 12.598	1.769 (−8.049, −1.073) *P* = 0.011	63.49 ± 11.921	52.00 ± 10.813	2.018 (−15.467, −7.509)	1.067 (1.028, 1.107) *P* < 0.01
Male, n (%)	44 (57)	70 (52)	1.238 (0.705, 2.175)	92 (54)	22 (52)	1.072 (0.545, 2.109)	NS
Carotid arteries plaques, n (%)	30 (39)	52 (39)	1.019 (0.574, 1.809)	76 (45)	6 (14)	4.851 (1.942, 12.120) *P* < 0.01	3.544 (1.307, 9.610) *P* < 0.05
Arterial hypertension, n (%)	48 (62)	96 (71)	0.672 (0.372, 1.216)	129 (76)	15 (36)	5.663 (2.750, 11.663) *P* < 0.01	5.427 (2.345, 12.559) *P* < 0.01
Diabetes, n (%)	28 (36)	55 (41)	0.831 (0.467, 1.481)	73 (43)	10 (24)	2.408 (1.113, 5.213) *P* < 0.05	1.218 (0.485, 3.054)
Dyslipidemia, n (%)	50 (65)	82 (61)	1.197 (0.669, 2.142)	103 (61)	29 (69)	0.689 (0.334, 1.420)	
CAD, n (%)	17 (22)	30 (22)	0.992 (0.505, 1.946)	40 (24)	7 (17)	1.538 (0.635, 3.730)	
Hyperhomocysteinemia, n (%)	27 (35)	35 (26)	1.543 (0.842, 2.828)	56 (33)	6 (14)	2.947 (1.173, 7.407) *P* < 0.05	2.683 (0.932, 7.724)
Hyperuricemia, n (%)	3 (4)	6 (4)	0.872 (0.212, 3.588)	7 (4)	2 (5)	0.859 (0.172, 4.293)	
Smoking, n (%)	23 (30)	36 (27)	1.171 (0.630, 2.176)	44 (26)	15 (36)	0.629 (0.306, 1.289)	
Alcoholism, n (%)	12 (16)	25 (19)	0.812 (0.382, 1.726)	31 (18)	6 (14)	1.338 (0.519, 3.453)	
Moderate or severe intracranial/extracranial arterial stenosis, n (%)	5 (6)	18 (13)	0.451 (0.161, 1.269)	20 (12)	3 (7)	1.733 (0.490, 6.133)	

*VBD indicates vertebrobasilar dolichoectasia; CSVD, cerebral small vessel disease; and CAD, coronary artery disease.*

### Serum MMP and TIMP Levels and Vertebrobasilar Arteries Parameters

Dilation was defined as the diameter of the BA being greater than 4.5 mm or of the VAs being greater than 4.0 mm. Patients exhibiting dilation (dilation group, *n* = 35) were directly compared to those with healthy BA and VA diameters (non-dilation group, *n* = 177). The MMP-9 serum level was significantly higher in the dilation group compared to the non-dilation group (*P* = 0.021). However, there was no significant difference in MMP-2, -3, TIMP-1, -2 or cathepsin L serum level between two groups (all *P* > 0.05). Further, the ratio of MMP-2/TIMP-2 level was not significantly different between groups, however, the ratio of MMP-9/TIMP-1 level was in significant difference (*P* = 0.010, Table 2).

**TABLE 2:** Matrix metalloproteinases between dilation and non-dilation of the basilar artery and vertebral arteries, and between bending and non-bending of the basilar artery.

	**Dilation (*n* = 35)**	**Non-dilation (*n* = 177)**	***P***	**Tortuosity (*n* = 16)**	**Non-tortuosity (*n* = 196)**	***P***	**Elongation (*n* = 55)**	**Non-elongation (*n* = 157)**	***P***
MMP-2	241.021 (64.251)	245.530 (87.473)	0.348	254.783 (58.221)	243.052 (82.154)	0.406	248.998 (69.514)	243.265 (83.230)	0.936
MMP-3	10.119 (5.910)	11.654 (10.855)	0.152	11.533 (7.026)	11.248 (9.818)	0.806	12.416 (12.434)	10.700 (9.176)	0.303
MMP-9	623.769 (400.392)	477.477 (405.039)	0.021	552.271 (492.722)	486.892 (408.959)	0.430	623.769 (380.041)	443.929 (406.197)	0.004
TIMP-1	174.334 (45.687)	179.298 (44.897)	0.455	195.404 (34.162)	175.107 (42.914)	0.039	186.285 (48.177)	175.065 (42.253)	0.210
TIMP-2	88.467 (14.940)	85.710 (20.428)	0.406	88.926 (12.714)	86.190 (20.163)	0.401	87.186 (19.153)	86.169 (19.645)	0.581
Cathepsin L	1907.068 (1033.838)	2141.813 (1173.889)	0.170	2598.747 (1477.333)	2035.976 (1119.841)	0.207	2244.540 (1211.156)	1969.576 (1107.815)	0.165
MMP-2/TIMP-2	2.658 (0.57)	2.916 (0.760)	0.087	2.789 (0.75)	2.861 (0.76)	0.656	2.750 (0.81)	2.895 (0.71)	0.857
MMP-9/TIMP-1	3.258 (2.25)	2.531 (1.89)	0.010	2.819 (2.38)	2.672 (2.20)	0.993	3.378 (2.18)	2.511 (1.90)	0.016

*MMP indicates matrix metalloproteinase; and TIMP, tissue inhibitor of metalloproteinase. MMP-2, 3, 9, TIMP-1 and -2 were expressed in *ng/ml* and cathepsin L was expressed in *pg/ml.**

Basilar artery tortuosity was defined as the BL being great than 8.0 mm; patients were divided into a tortuosity group (*n* = 16) or a non-tortuosity group (*n* = 196) based on this criterion. TIMP-1 serum level was markedly higher in the tortuosity group (*P* = 0.039) than the non-tortuosity group. Conversely, the level of MMP-2, -3, -9, TIMP-2 and cathepsin L serum was similar in the two groups (all *P* > 0.05, Table 2). The ratio of MMP-2/TIMP-2 level or MMP-9/TIMP-1 level were neither significantly different between groups (*P* > 0.05, Table 2).

Patients were also divided into an elongation group (*n* = 55) or a non-elongation group (*n* = 157) according to the BAL. MMP-9 serum level was significantly higher in the elongation group compared to the non-elongation group (*P* = 0.004). However, there was no significant difference in MMP-2, -3, TIMP-1, -2 or cathepsin L serum levels between two groups (all *P* > 0.05, Table 2). The ratio of MMP-2/TIMP-2 level was not significantly different between groups, however, the ratio of MMP-9/TIMP-1 level was in significant difference (*P* = 0.016, Table 2).

**FIGURE 5:**
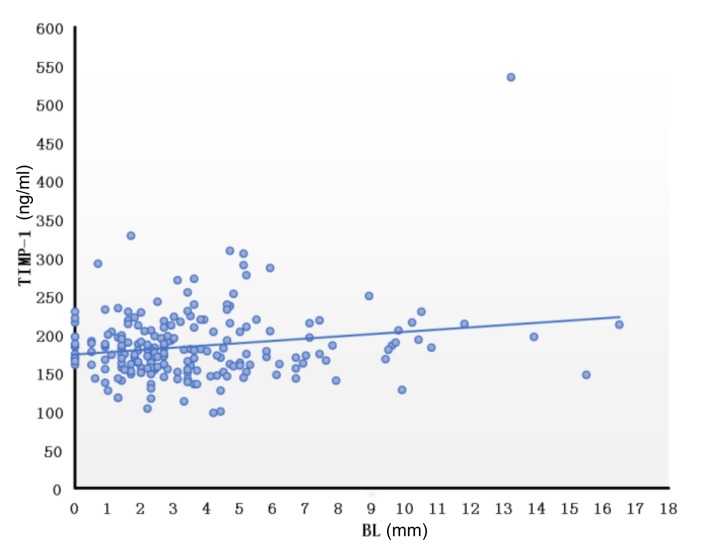
Correlation of bending length of the basilar artery and TIMP-1. Correlation between BL and TIMP was calculated using the Pearson correlation coefficient after controlling for age and gender (*r* = 0.161, *P* = 0.020). BL, bending length; TIMP, tissue inhibitor of metalloproteinase.

We further calculated the correlation between serum MMPs and TIMPs levels and VA parameters. We found that the maximum diameter of the right VA was negatively correlated with MMP-2 serum level (*r* = -0138, *P* = 0.046) and positively correlated with MMP-9 serum level (*r* = 0.164, *P* = 0.018). Furthermore, TI was positively correlated with TIMP-1 serum level (*r* = 0.142, *P* = 0.041). The diameter or TI of the left VA was not correlated with any MMP or TIMP levels. Interestingly, we found that the BL of the BA was positively correlated with TIMP-1 serum level (Figure 5) (*r* = 0.161, *P* = 0.020) though the diameter, BAL and TI of the BA were not correlated with any MMP or TIMP serum level. The parameters of VAs and BA were not correlated with the ratio of MMP-2/TIMP-2 level or MMP-9/TIMP-1 level (*P* > 0.05).

### Vertebrobasilar Arteries Parameters and the Neuroimaging Markers of Cerebral Small Vessel Disease

To investigate the association between VBD and CSVD, neuroimaging parameters including the diameters and TI of the VAs and BA, as well as the BAL and BL of the BA were evaluated between vertigo patients with or without LI and among different grades of WMH. The diameter and TI of the VAs and BA, and the BAL and BL of the BA were all similar in vertigo patients with LI (*n* = 27) and those without LI (*n* = 185) (Table 3). The diameter of the VAs and BA were similar in vertigo patients with different WMH grades. Interestingly though, the BAL, TI and BL were significantly greater (all *P* < 0.01) in vertigo patients with higher WMH grades (grade 2 and 3) (*n* = 85) than those with lower WMH grades (grade 0 and 1) (*n* = 127) (Table 3). We also found that the TI of the VAs were nearly significantly greater (both *P* < 0.20) in high grade WMH patients compared to low grade WMH patients (Table 3).

**TABLE 3:** Association between cerebral small vessel disease and vertebrobasilar artery parameters and MMPs.

	**LI (*n* = 27)**	**Non-LI (*n* = 185)**	***P***	**WMH**				***P***
					
				**Grade 0 (*n* = 48)**	**Grade 1 (*n* = 79)**	**Grade 2 (*n* = 57)**	**Grade 3 (*n* = 28)**	
**Vertebrobasilar arteries parameters**								
Left vertebral artery								
Diameter	2.90 (1.60)	2.90 (1.20)	0.668	3.10 (1.20)	3.15 (1.20)	2.80 (1.30)	2.80 (1.50)	0.791
TI	0.958 (0.105)	0.967 (0.099)	0.514	0.769 (0.112)	0.101 (0.094)	0.103 (0.104)	1.116 (0.105)	0.072
Right vertebral artery								
Diameter	2.90 (1.70)	2.50 (1.20)	0.078	2.45 (1.30)	2.50 (1.40)	2.60 (1.20)	2.75 (1.30)	0.403
TI	0.739 (0.038)	0.657 (0.061)	0.369	0.050 (0.060)	0.067 (0.044)	0.070 (0.064)	0.068 (0.053)	0.161
Basilar artery								
Diameter	3.37 (0.90)	3.27 (0.80)	0.285	3.15 (0.90)	3.35 (0.80)	3.30 (0.70)	3.35 (1.40)	0.631
BAL	26.30 (6.80)	26.35 (6.10)	0.490	26.25 (5.30)	25.55 (6.00)	26.70 (8.40)	30.85 (11.10)	0.000
TI	0.056 (0.100)	0.045 (0.087)	0.362	0.054 (0.055)	0.031 (0.071)	0.042 (0.126)	0.112 (0.200)	0.001
BL	3.40 (3.70)	2.70 (3.00)	0.493	2.85 (2.70)	2.30 (2.20)	2.60 (3.60)	4.65 (6.90)	0.000
**MMPs parameters**								
MMP-2	261.736 (74.064)	242.879 (82.154)	0.421	243.545 (100.032)	242.879 (81.825)	257.069 (85.350)	242.646 (39.044)	0.769
MMP-3	12.728 (15.328)	11.195 (8.982)	0.294	12.041 (11.551)	9.924 (8.861)	11.713 (10.967)	10.564 (7.465)	0.388
MMP-9	564.603 (479.015)	481.822 (389.665)	0.323	428.596 (389.421)	496.223 (496.223)	562.006 (394.830)	514.535 (363.744)	0.392
TIMP-1	189.834 (52.559)	175.107 (45.541)	0.098	161.035 (37.367)	175.461 (41.415)	184.360 (39.317)	187.950 (62.638)	0.008
TIMP-2	89.7129 (16.449)	85.960 (20.039)	0.507	89.676 (19.469)	84.368 (23.227)	88.467 (17.197)	86.698 (14.788)	0.622
Cathepsin L	2244.540 (1070.235)	2030.142 (1177.967)	0.178	1830.532 (903.881)	2175.154 (1213.089)	2236.315 (1340.341)	1960.427 (1356.551)	0.150
MMP-2/TIMP-2	2.895 (0.70)	2.845 (0.73)	0.784	2.843 (0.72)	2.910 (0.84)	2.916 (0.65)	2.734 (0.76)	0.946
MMP-9/TIMP-1	3.091 (2.62)	2.668 (2.19)	0.558	2.486 (2.33)	2.668 (2.46)	2.695 (2.17)	2.819 (1.70)	0.872

*LI indicates lacunar infarction; WMH, white matter hyperintensities; TI, tortuosity index; BAL, basilar artery length; BL, bending length; MMP, matrix metalloproteinase; and TIMP, tissue inhibitor of metalloproteinase.*

### Serum MMP and TIMP Levels and the Neuroimaging Markers of Cerebral Small Vessel Disease

The neuroimaging markers of CSVD, including LI and WMH, were observed in the current study. There was no significant difference in the serum level of MMP-2, -3, -9, TIMP-2 or cathepsin L, or the ratio of MMP-2/TIMP-2 level or MMP-9/TIMP-1 level between vertigo patients with LI (*n* = 27) and those without LI (*n* = 185). We did, however, identify a near significant difference in the TIMP-1 serum level, being higher in vertigo patients with LI compared to those without LI (*P* < 0.10, Table 3). On the other hand, the TIMP-1 serum level was markedly higher in vertigo patients with high grade WMH (grade 2 and 3) (*n* = 85) than those with low grade WMH (grade 0 and grade 1) (*n* = 127) (*P* < 0.01). There was no significant difference in the serum level of MMP-2, -3, -9, TIMP-2 or cathepsin L, or the ratio of MMP-2/TIMP-2 level or MMP-9/TIMP-1 level among individuals with different WMH grades (Table 3).

## Discussion

The relationships between VBD and the imbalance of MMPs and TIMPs have been speculated for years, but it has not been clarified how the imbalance between MMPs and TIMPs play a role in the development and progression of dolichoectasia (Gutierrez et al., 2011; Pico et al., 2015). We found that MMP-9 serum level was higher in vertigo patients with VBA dilation and BA elongation, and the ratio of MMP-9/TIMP-1 level were higher in those patients, while TIMP-1 serum level was higher in those patients with BA tortuosity. The BL, which represents the degree of BA tortuosity, was positively correlated with TIMP-1. MMP-9 and TIMP-1 serum levels were both higher in vertigo patients with VBD, and we further found that upregulation of MMP-9 might be accompanied by the downregulation of TIMP-1 but not by TIMP-2 regulation.

Matrix metalloproteinases and TIMPs maintain a relative balance in normal tissues (Cui et al., 2017) and as this balance is disturbed, the degradation of extracellular matrix occurs. A recent study on patients with Graves’ orbitopathy unfolded that MMP-2, MMP-9, TIMP-1 and TIMP-2 serum concentrations were all significantly higher than healthy controls (Kapelko-Słowik et al., 2018). In addition, another study on multiple myeloma patients revealed that MMP-2 and MMP-9 are secreted in higher amounts and are not balanced by inhibitors of TIMPs (Urbaniak-Kujda et al., 2016). Therefore, growing evidence supports the phenomenon that MMPs and TIMPs maintain a balance in a normal state, and when broken, both MMPs and TIMPs are regulated via a positive feedback loop. For example, increasing MMPs breaks the balance between MMPs and TIMPs, and the body compensates by increasing TIMPs via a positive feedback mechanism. However, if the MMP increase surpasses the capability of such regulation, increasing TIMPs is inadequate to regain balance with MMPs. This can lead to the degradation of extracellular proteins located in the tunica media, such as elastin, collagen, or proteoglycans, leading to the procession of artery dilation (Pico et al., 2015). Nevertheless, how excessive increases of TIMPs lead to the dolichoectasia of arteries remains unclear. In the current study, MMP-9 and TIMP-1 serum levels were both higher in patients with VBD, and the ratio of MMP-9/TIMP-1 was higher in patients with vertebrobasilar dilation and elongation, which was likely to add the evidence that increasing MMP-9 beaks the balance between MMP-9 and TIMP-1 leading to the dilation or elongation of arteries. However, the interesting phenomenon of the balance between MMPs and TIMPs in vertigo patients with VBD should be further evaluated in future study, especially evaluating an animal model with genetic or pharmacological tools, to elucidate a deeper understanding of pathophysiological VBD.

We also found that CSVD was more common in vertigo patients with VBD than those without VBD. Very recent studies have revealed that CSVD is frequent in stroke patients with intracranial arteries dolichoectasia (Thijs et al., 2017; Zhai et al., 2018; Fierini et al., 2019). Importantly, we evaluated VBD with quantitative image parameters, and found that BAL, and the BL and the TI of the BA were markedly greater (and the TI of the VAs to a lesser extent) in vertigo patients with higher WMH grades (grade 2 and 3) compared to those with lower WMH grades (grade 0 and 1). Similarly, a new report (Thijs et al., 2017) also found that dolichoectasia was associated with higher grades of WMH, however, they didn’t assess the degree of VBD. CSVD is an arteriopathy affecting the media of the artery as well, and it is associated with MMP and TIMP levels (Rosenberg et al., 2001; Zhang et al., 2015; Del Brutto et al., 2017). A study discovered that microglia/macrophages cells contained MMP-3 were more present around small perforating arteries and areas of severe white matter damage corresponding to leukoaraiosis (Rosenberg et al., 2001). Another study concluded that MMP-2-1306 T/C polymorphism was associated with moderate or severe leukoaraiosis (Zhang et al., 2015). However, in the current study, MMP-2, -3 or -9 levels were not higher in vertigo patients with LI or advanced stages of WMH, whereas the TIMP-1 level was higher in vertigo patients with higher WMH grades (grade 2 and 3). Our results, to some extent, support the hypothesis that the imbalance between MMPs and TIMPs could be a bridge between VBD and WMH. However, in order to provide mechanistic evidences, an animal model with genetic or pharmacological tools should be well performed in a further series of studies to provide hard evidence of the causality relationship among MMPs, TIMPs, VBD and WMH.

Some limitations in our study need consideration. First, this study was a cross-sectional study in a single center and asymptomatic patients were excluded. Thus, our data were limited, and a selection bias might be unavoidable. Second, recognizing the dilated perivascular spaces was subjective and may have led to large heterogeneity across patients. Furthermore, there were just three participating patients that underwent susceptibility-weighted imaging to detect cerebral microbleeds, and we failed to evaluate these neuroimaging markers of CSVD in our study. Third, we evaluated MMP and TIMP serum levels but never performed the pathological examination to evaluate the local concentrations of MMPs and TIMPs in the VBAs or in the areas of LI or WMH.

## Conclusion

In conclusions, an imbalance between MMP-9 and TIMP-1 levels probably link the extent of VBD and WMH in the current study. An upregulation of MMP-9 mainly might result in VBA dilation of the VAs and elongation of the BA while an upregulation of TIMP-1 mainly might lead to BA tortuosity. This BA tortuosity likely contributes to the occurrence and progression of WMH. Future studies will expand the sample size and conduct regular patient follow-ups to test our hypothesis. An animal model with genetic or pharmacological tools also should be evaluated to provide hard evidence on the association among VBD, CSVD and MMPs and TIMPs.

## Data Availability

All datasets generated for this study are included in the manuscript and/or the supplementary files.

## Author Contributions

DZ and MZ conceived this study and provided financial support. YP analyzed the whole data and wrote the draft manuscript. HZ and JG collected the clinical data of the patients. SY collected and analyzed the image data. TW tested the serum levels of MMPs and TIMPs.

## Conflict of Interest Statement

The authors declare that the research was conducted in the absence of any commercial or financial relationships that could be construed as a potential conflict of interest.
